# Reversible uptake of molecular oxygen by heteroligand Co(II)–l-α-amino acid–imidazole systems: equilibrium models at full mass balance

**DOI:** 10.1186/s13065-017-0319-8

**Published:** 2017-09-19

**Authors:** Marek Pająk, Magdalena Woźniczka, Andrzej Vogt, Aleksander Kufelnicki

**Affiliations:** 1 Department of Physical and Biocoordination Chemistry, Faculty of Pharmacy, Medical University of Łódź, Muszyńskiego 1, 90-151 Lodz, Poland; 2 Faculty of Chemistry, University of Wrocław, F. Joliot-Curie 14, 50-383 Wrocław, Poland

**Keywords:** Co(II), l-α-amino acid, Imidazole, Dioxygen, Oxygen complex, $K_{{{\text{O}}_{2} }}$ equilibrium constant, Mass balance

## Abstract

**Background:**

The paper examines Co(II)–amino acid–imidazole systems (where amino acid = l-α-amino acid: alanine, asparagine, histidine) which, when in aqueous solutions, activate and reversibly take up dioxygen, while maintaining the structural scheme of the heme group (imidazole as axial ligand and O_2_ uptake at the sixth, trans position) thus imitating natural respiratory pigments such as myoglobin and hemoglobin. The oxygenated reaction shows higher reversibility than for Co(II)–amac systems with analogous amino acids without imidazole. Unlike previous investigations of the heteroligand Co(II)–amino acid–imidazole systems, the present study accurately calculates all equilibrium forms present in solution and determines the $K_{{{\text{O}}_{2} }}$equilibrium constants without using any simplified approximations. The equilibrium concentrations of Co(II), amino acid, imidazole and the formed complex species were calculated using constant data obtained for analogous systems under oxygen-free conditions. Pehametric and volumetric (oxygenation) studies allowed the stoichiometry of O_2_ uptake reaction and coordination mode of the central ion in the forming oxygen adduct to be determined. The values of dioxygen uptake equilibrium constants $K_{{{\text{O}}_{2} }}$ were evaluated by applying the full mass balance equations.

**Results:**

Investigations of oxygenation of the Co(II)–amino acid–imidazole systems indicated that dioxygen uptake proceeds along with a rise in pH to 9–10. The percentage of reversibility noted after acidification of the solution to the initial pH ranged within ca 30–60% for alanine, 40–70% for asparagine and 50–90% for histidine, with a rising tendency along with the increasing share of amino acid in the Co(II): amino acid: imidazole ratio. Calculations of the share of the free Co(II) ion as well as of the particular complex species existing in solution beside the oxygen adduct (regarding dioxygen bound both reversibly and irreversibly) indicated quite significant values for the systems with alanine and asparagine—in those cases the of oxygenation reaction is right shifted to a relatively lower extent. The experimental results indicate that the “active” complex, able to take up dioxygen, is a heteroligand CoL_2_L′complex, where L = amac (an amino acid with a non-protonated amine group) while L′ = Himid, with the N1 nitrogen protonated within the entire pH range under study. Moreover, the corresponding log  $K_{{{\text{O}}_{2} }}$ value at various initial total Co(II), amino acid and imidazole concentrations was found to be constant within the limits of error, which confirms those results. The highest log $K_{{{\text{O}}_{2} }}$ value, 14.9, occurs for the histidine system; in comparison, asparagine is 7.8 and alanine is 9.7. This high value is most likely due to the participation of the additional effective N3 donor of the imidazole side group of histidine.

**Conclusions:**

The Co(II)–amac–Himid systems formed by using a [Co(imid)_2_]_n_ polymer as starting material demonstrate that the reversible uptake of molecular oxygen occurs by forming dimeric μ-peroxy adducts. The essential impact on the electron structure of the dioxygen bridge, and therefore, on the reversibility of O_2_ uptake, is due to the imidazole group at axial position (trans towards O_2_). However, the results of reversibility measurements of O_2_ uptake, unequivocally indicate a much higher effectiveness of dioxygenation than in systems in which the oxygen adducts are formed in equilibrium mixtures during titration of solutions containing Co(II) ions, the amino acid and imidazole, separately.

**Electronic supplementary material:**

The online version of this article (doi:10.1186/s13065-017-0319-8) contains supplementary material, which is available to authorized users.

## Background

The capability of compounds called natural respiratory pigments to reversibly absorb molecular oxygen has been the subject of intensive research since the end of the 19th Century and has been inspiring the creation of artificial systems to imitate their activity [1–14]. Example models of synthetic oxygen carriers include mixed complexes of the type Co(II)–auxiliary ligand–imidazole, in which imidazole coordinates in trans position against the bound O_2_ molecule, alike imidazole of the proximal histidine in myoglobin and hemoglobin [15]. In contrast to classical methods of preparing such compounds by mixing separate solutions of Co(II) salts, appropriate amino acids and imidazole [16–18], an original method has been applied, in which cobalt(II) and imidazole were introduced in the form of a polymeric, pseudo-tetrahedral, semi-conductive complex [Co(imid)_2_]_n_. This results in the formation of definite, unique structures with an imidazole molecule in an axial position opposite the O_2_ molecule [19–26].

**Fig. 1:**
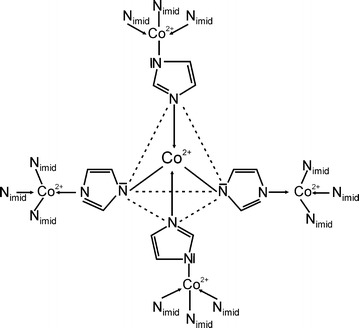
Schematic structure of the polymeric [Co(imid)_2_]_n_ complex

[Co(imid)_2_]_n_ is a coordination compound crystallizing in an infinite polymeric net, in which each cobalt(II) ion is joined via imidazole bridges with four adjacent ions of the metal [27, 28]. Each Co(II) ion forms two dative bonds with the nitrogen atoms of two deprotonated imidazole moieties and two ionic bonds with the nitrogen atoms of two other imidazoles (Fig. 1). Therefore, this alternative method of obtaining dioxygen complexes with a strictly defined structure by starting from the [Co(imid)_2_]_n_ polymer is much more effective than the method in which appropriate so-called “active” complexes capable of reversible dioxygen uptake are formed in an equilibrium mixture during titration of a solution containing Co(II) ions, the suitable auxiliary ligand (e.g. amino acid) and imidazole [16, 17].

The peculiar property of O_2_ transport in such Co(II)–amac–Himid systems, as with the natural dioxygen carriers, results from the rapidly stabilizing equilibrium present in solution between the “active” form and the dioxygen-containing form. The “active” form, responsible for the dioxygen transport, is usually a paramagnetic, high-spin, hexacoordinate Co(II) complex of Co^II^(amac)_2_(Himid)(H_2_O) composition, containing two chelate–like connected amino acid molecules forming an equatorial plane, as well as two axial ligands–imidazole and water. After substitution of the dioxygen molecule for water, a dimeric, diamagnetic [Co^III^(amac)_2_(Himid)]_2_O_2_^2−^ complex is formed with the O_2_ molecule coordinated in peroxide order i.e. with a O_2_^2−^ (μ-peroxy) bridge between two cobalt ions formally oxygenated to Co(III). This complex, because of the eventual partial irreversible oxidation of Co(II) to mononuclear Co(III) products, is frequently denoted as an intermediate oxygen adduct. Owing to the elongation of the dioxygen bond from 120.7 pm for the triplet O_2_ to 149.0 pm for the peroxide O_2_^2−^ anion, the oxygen adducts may be used as intermediate complexes in catalytic processes [29–34].

The O_2_^2−^ bridge (μ-peroxy) exists within pH = 3–9, but upon a rise in basicity above pH 10, this is transformed into a poorly reversible dibridged Co(III)O_2_^2−^OH^−^Co(III) (μ-peroxy–μ-hydroxy) form. This double-bridge appears in place of the two carboxyl groups, which easily undergo dissociation and which are found in *cis* position towards the coordinated dioxygen molecule. Such a complex is a much less effective O_2_ carrier due to its higher affinity for autoxidation. An alternative known description of the oxygen bridges is the form type *η*, corresponding to “side on” bridge μ-peroxy structures [35]. In turn, acidification of the solution at a low temperature (−3 to 0 °C) leads to protonation of the μ-peroxy bridge, whereas the forming intermediate Co(III)O_2_^2−^H^+^Co(III) product undergoes a rapid decay accompanied by Co(III) ion formation. In addition, at a temperature around 0 °C and in acidic medium, the O_2_^2−^ (μ-peroxy) bridge may be subsequently oxidized by means of strong oxidizers, e.g. Ce^4+^, MnO_4_^−^ or Cl_2_ ions. As a result, a paramagnetic, stable {[Co^III^(amac)_2_(Himid)]_2_O_2_^−^}^+^ complex is formed, with an irreversibly bound dioxygen moiety in the Co(III)–O_2_^−^–Co(III) (μ-superoxy) bridge.

All known O_2_ carriers (both natural and synthetic) form complexes of two types: monomeric, with an M:O_2_ stoichiometry of 1:1, and dimeric, with an M:O_2_ stoichiometry of 2:1. An analysis of the theoretically estimated values of the free standard Gibbs energy of the O_2_ reactions with metal ions and their complexes could be expected to favor the dimeric structures. In fact, the Δ*G*° value for the dimer formation reaction attains negative values for a much higher number of metals than is the case for monomer formation. This effect refers to the displacement of complex-formation decidedly to the right [36]. The data find a practical confirmation because among all the known dioxygen carriers, in aqueous solution we observe formation of stable dimeric complexes.

Previous investigations of the Co(II)–amac–Himid systems have not included the key aspect, i.e. accurate calculations of the Co(II), amac and Himid concentrations at equilibrium, by using the formation constants reported in our work for analogous oxygen-free systems [37]. These calculations may allow the equilibrium concentrations of all equilibrium forms present in solution to be determined, and for the $K_{{{\text{O}}_{2} }}$ equilibrium constants to be evaluated without using any simplified approximations, which for instance take into account only the “active” complex and the oxygen adduct within the mass balance system [19, 38]. Moreover, the advantage of the experimental methods used in the present work, i.e. a direct gas–volumetric experiment with simultaneous pH measurement, is that it allows the degree of reversibility of O_2_ uptake to be taken into account. As for many other complexes, including a majority of complexes with amino acids and peptides, the irreversible part of the reaction is quite rapid (e.g. t_1/2_ <5 min for glygly), which excludes the use of the most commonly applied method based only on potentiometric titration [39–41].

## Results and discussion

The optimum amac to Co(II) ratio equaled 2:1. Above this value, the amount of dioxygen taken up did not change (see Additional file 1: Figure S1). The amount of imidazole released from the [Co(imid)_2_]_n_ moiety as a result of the mixed Co(II)–amac–Himid–O_2_ complex formation (0.3 mol Himid per 0.3 mol Co) indicates that the stoichiometric Co(II): imidazole ratio was 1:1, which confirms that one of the two [Co(imid)_2_]_n_ imidazole moieties remains in the coordination sphere of cobalt(II) of the final complex (see Additional file 2: Figure S2). In other words the structure of the forming dioxygen adducts are unified by the presence of one imidazole in the coordination sphere.

**Fig. 2:**
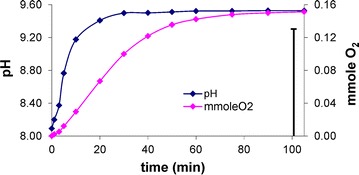
The Co(II)–l-α-histidine–Himid system at molar ratio 0.3:0.75:0.3 (mmol). Dependence of pH and number of mmole O_2_ bound on duration of the oxygenation reaction at a temperature of ~0°C (vertical segment corresponds to reversibility of O_2_ uptake after saturation = 86.69%)

Investigations of oxygenation of the Co(II)–amac–Himid systems indicated that dioxygen uptake is accompanied by a rise in pH to 9–10. An example of the time dependence between pH and the number of mmoles of bound dioxygen is shown for l-α-histidine in Fig. 2. The percentage of reversibility noted after acidification of the solution to the initial pH ranged within ca 30–60% for l–α–alanine, 40–70% for l-α-asparagine and 50–90% for l-α-histidine; this rose as the share of the amino acid in the Co(II): amac: Himid ratio increased (Table 1). The results confirm that the axial imidazole plays a role in enhancing reversibility of the O_2_ uptake as opposed to the systems with the same amino acids but lacking imidazole [42]. Imidazole is in fact an important complement of the coordination sphere of the central ion as a donor of a free-electron pair of the N3 nitrogen.

**Table 1:** Uptake of O_2_ by the Co(II)–l-α-amino acid–imidazole systems (duration time of uptake, final value of pH, number of mmoles of O_2_ bound, percentage of reversibility)

Amac	Co(II): amac:Himid (mmole ratio)	Time (min)	pH	mmoles O_2_	% revers
Alanine	0.3: 0.15:0.3	90	9.882	0.0515	30.33
	0.3:0.225:0.3	105	9.557	0.0559	31.25
	0.3:0.3:0.3	210	9.751	0.0918	31.80
	0.3:0.375:0.3	215	9.629	0.0936	46.76
	0.3:0.5:0.3	215	9.562	0.1193	57.71
	0.3:0.6:0.3	270	9.603	0.1343	49.68
	0.3:0.75:0.3	195	9.418	0.1457	45.56
	0.3:0.9:0.3	270	9.422	0.1661	46.65
	0.3:1.05:0.3	200	9.276	0.1690	48.97
	0.3:1.2:0.3	120	9.247	0.1704	57.72
Asparagine	0.3:0.15:0.3	210	9.498	0.0352	43.37
	0.3:0.225:0.3	150	9.752	0.0567	45.04
	0.3:0.3:0.3	270	9.634	0.0600	44.37
	0.3:0.375:0.3	190	9.656	0.0936	49.77
	0.3:0.45:0.3	150	9.543	0.0901	53.55
	0.3:0.6:0.3	120	9.152	0.1219	60.78
	0.3:0.75:0.3	120	8.919	0.1237	57.84
	0.3:0.9:0.3	200	9.022	0.1304	65.27
	0.3:1.2:0.3	180	8.616	0.1275	67.67
Histidine	0.3:0.15:0.3	90	9.668	0.0373	51.16
	0.3:0.225:0.3	70	9.687	0.0565	52.85
	0.3:0.3:0.3	150	9.535	0.0755	72.99
	0.3:0.375:0.3	70	9.412	0.0932	68.98
	0.3:0.45:0.3	150	9.732	0.1123	69.81
	0.3:0.6:0.3	90	9.193	0.1496	82.63
	0.3:0.75:0.3	105	9.526	0.1513	86.69
	0.3:0.9:0.3	90	9.345	0.1502	85.06
	0.3:1.2:0.3	90	9.234	0.1507	84.81

**Fig. 3:**
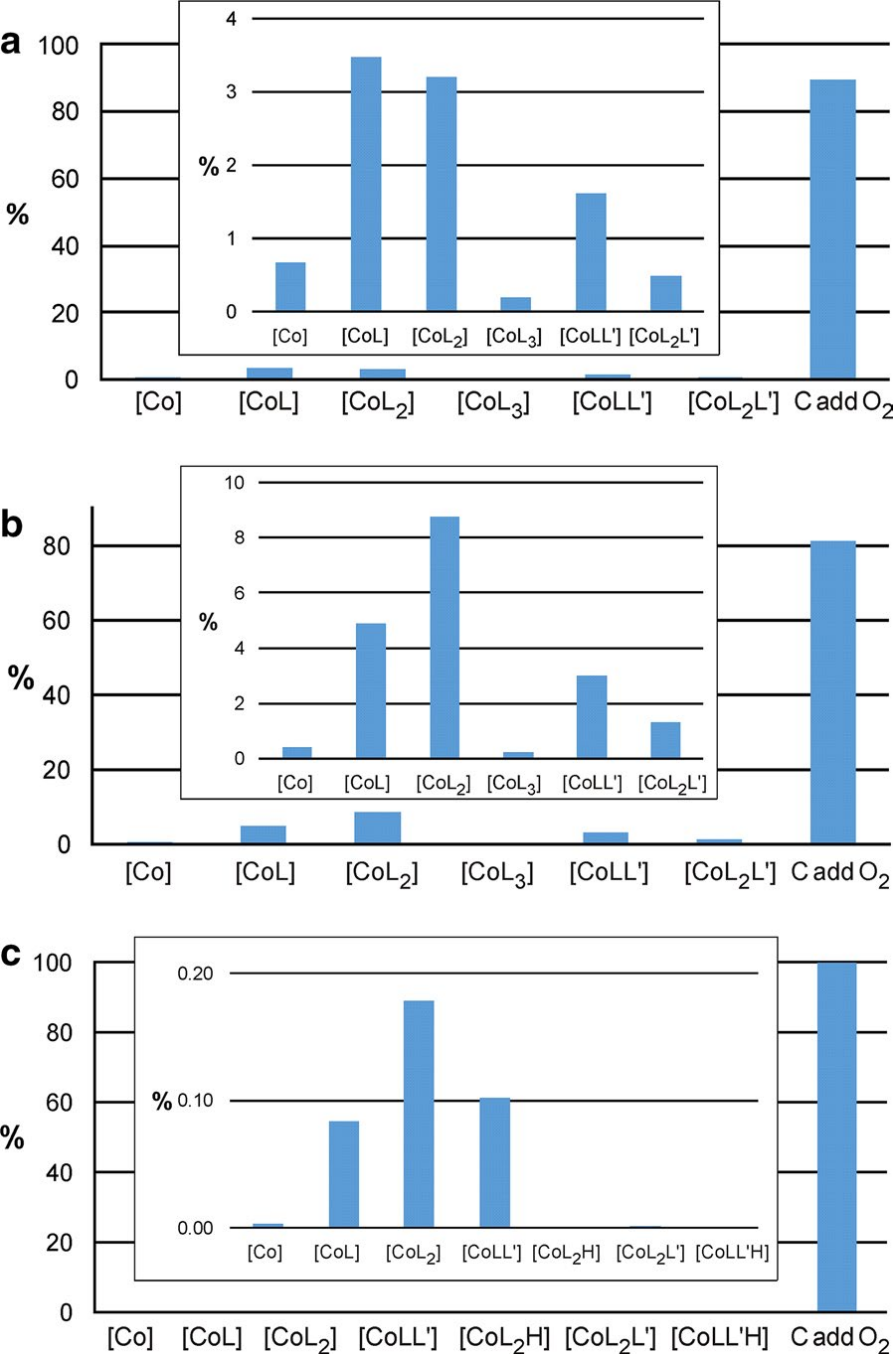
Percentage share of free Co(II) ion and complex forms at fixed equilibrium of O_2_ uptake reaction in the Co(II)–amac–Himid–O_2_ systems. l-α-amino acid (amac) = **a** alanine, **b** asparagine, **c** histidine. Internal diagrams show the equilibrium share of species other than the O_2_ adduct in an extended scale. C_add_ O_2_ denotes the total concentration of the dioxygen adduct(with dioxygen bound both reversibly and irreversibly). L amac, L′ Himid. Molar ratio Co(II): amac: Himid = 0.3: 0.6:0.3 (mmol)

**Fig. 4:**
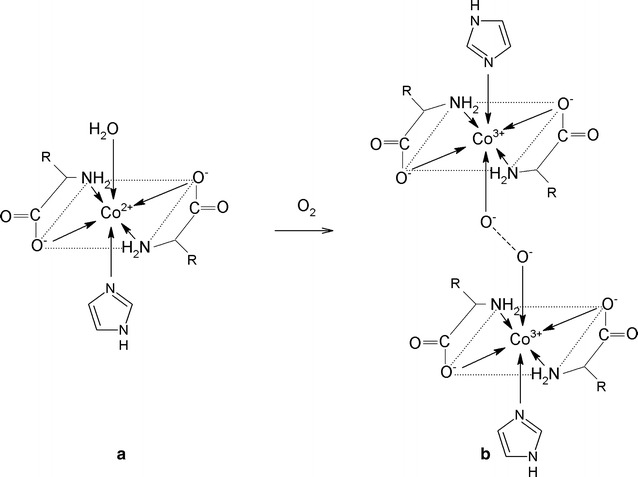
Coordination modes in the Co(II)–amac–Himid system with amac = Ala (R = CH_3_) and Asn (R = CH_2_–CO–NH_2_). **a** “active” heteroligand complex CoL_2_L′, where L amac, L′ Himid, **b** dioxygen adduct

**Fig. 5:**
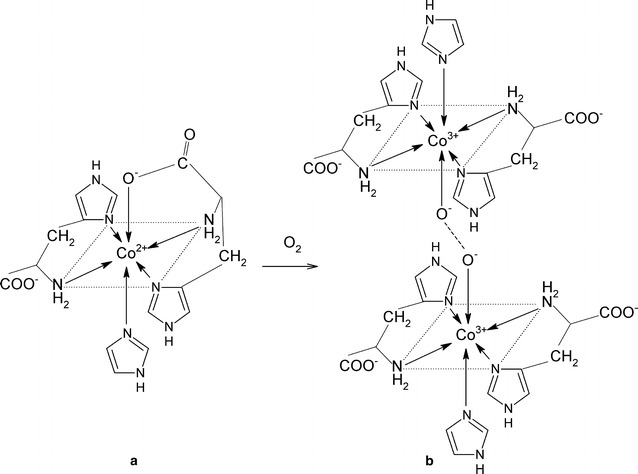
Coordination modes in the Co(II)–amac–Himid system with amac = His. **a** “active” heteroligand complex CoL_2_L′, where L amac, L′ Himid, **b** dioxygen adduct

In comparison with the system with histidine, the systems with alanine and asparagine demonstrated some higher values for the share of the free Co(II) ion and the particular complex species existing in solution apart from the oxygen adduct (regarded as the entire amount of cobalt engaged in both reversible and irreversible oxygenation) Fig. 3. For the two amino acids, the oxygenation reaction is right shifted to a relatively lower extent. The competitive binary complexes CoL_3_ (or CoL_2_ for histidine), which are able to reversibly take up dioxygen, are also present in solution in relatively low concentrations below 0.2% (Fig. 3); according to Fallab’s rule, they have a sufficient number of 3N in the coordination sphere [40]. However, the experimental results indicate that the only active complexes taking up dioxygen in practice are heteroligand species with imidazole as the second ligand (Additional file 2: Figure S2). Therefore, the $K_{{{\text{O}}_{2} }}$ equilibrium constants can be calculated using formula (16), where the “active” complex is an appropriate heteroligand species with a concentration directly following the full mass balance equation. The fact that the value of log  $K_{{{\text{O}}_{2} }}$ remained constant between different initial total Co(II), amino acid and imidazole concentrations, within limits of error (Table 2), indicates that the “active” complex was a heteroligand CoL_2_L′complex for alanine and asparagine (Fig. 4), but also for histidine although the structure differs in participation of the N3 nitrogens of the additional imidazole side group (Fig. 5). The imidazole N1–H side-group does not dissociate in the measurable pH range due to it having a p*K* of 14.4 [43]. In addition, for histidine, as in the case of alanine and asparagine, the dioxygen substitutes a relatively weak donor, i.e. the deprotonated carboxyl oxygen, instead of the water molecule.

**Table 2:** Equilibrium constants $K_{{{\text{O}}_{2} }}$ of dioxygen uptake in the Co(II)–l-α-amino acid–imidazole–O_2_ systems

Amac	Co(II):amac:Himid (mmole ratio)	Log $K_{{{\text{O}}_{2} }}$
Alanine	0.3:0.5:0.3	9.442 10.957 10.216
	0.3:0.6:0.3	8.348 9.200 8.650
	0.3:0.75:0.3	10.061 10.107 10.100
Mean: 9.7 ± 0.8		
Asparagine	0.3:0.45:0.3	7.377 8.739 7.996
	0.3:0.6:0.3	7.520 8.246 7.959
	0.3:0.75:0.3	7.609 7.831 7.743
	0.3:0.9:0.3	7.371 7.593 7.439
	0.3:1.2:0.3	7.952 7.997 7.978
Mean: 7.8 ± 0.4		
Histidine	0.3:0.15:0.3	15.050 15.422 15.425
	0.3:0.375:0.3	14.002 14.175 14.179
	0.3:0.45:0.3	15.585 15.712 15.714
	0.3:0.6:0.3	14.537 14.471 14.475
Mean: 14.9 ± 0.7		

The mean values are followed by standard deviations

**Fig. 6:**
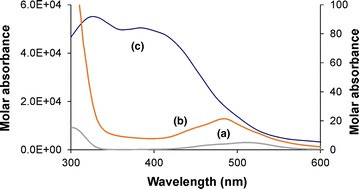
UV/Vis spectra in the Co(II)–amac–Himid system at temperature ~0 °C, where amac = l-α-histidine. Right Y-axis: molar absorption coefficients of (a) Co(II) and (b) “active” heteroligand complex CoL_2_L′, where L amac, L′ Himid; left Y-axis: (c) molar absorption coefficients of the dioxygen adduct

The optical absorption spectra for the Co(II)–amac–Himid system with histidine indicate a significant increase of the molar absorption coefficients resulting from the O_2_ uptake (Fig. 6); similar observations have been reported for analogous systems with alanine and asparagine [38]. The low energetic asymmetric *d*–*d* band in curve (a) can be attributed to the asymmetric, quasi-octahedral *T*_1g_→*T*_1g_(P) transition of the Co(H_2_O)_6_^2+^ aquo-ion. Curve (b) is a spectral curve mainly characterizing the formed heteroligand CoL_2_L′ active complex, predominating at pH ~9 under oxygen-free conditions, with a blue-shifted *d*–*d* band at *λ*_max_ 485 nm (*ε*_max_ ~20). Curve (c) corresponds to a μ-peroxo-type dioxygen adduct with two components of the LMCT band from the split antibonding π*(O_2_) orbital of dioxygen to the unfilled *d*σ*(Co) orbital: π*_h_ → *d*σ* (in-plane) and π*_v_→*d*σ* (out-of-plane). It can be seen that the molar absorption coefficient of both the bands (*ε*_max_ ~5 × 10^4^) is much higher than that of the “active” complex. The intensity of the two LMCT components was relatively comparable, this being typical of monobridged peroxo complexes, which are usually non-planar [38, 44].

As can be seen in Table 2, the highest value of log $K_{{{\text{O}}_{2} }}$ occurs for the histidine system, most likely due to participation of the aforementioned additional effective N3 donor of the histidine imidazole side group. This is not surprising as it is already known that for histidine, the “active” complex is the most thermodynamically stable complex also under oxygen-free conditions [37]. On the other hand, the lower value of log  $K_{{{\text{O}}_{2} }}$ for the asparagine system in comparison with the alanine system is most likely due to steric hindrance, which arises from one of the asparagine amide side groups during formation of the dimer. In this case, a greater share of the amino acid in the Co(II): amac: Himid molar ratio favorably displaces the equilibrium towards oxygen adduct formation. This also makes it possible to obtain chemically reasonable (positive) solutions of equation system (1) at higher excesses of the amino acid (cf. Table 2). For the two remaining amino acids alanine and histidine, particularly histidine, the oxygen adduct (for both the reversible and irreversible parts together) almost entirely uses up the accessible cobalt when the share of the amino acid greatly exceeds the stoichiometric ratio Co(II): amac:Himid = 1:2:1; the concentrations of the other complex species, including the “active” complex, fall to such low levels that it is impossible for the equation system (1) to converge in the form of three positive solutions.

## Conclusions

At a decreased temperature close to 0 °C, the Co(II)–amac–Himid systems demonstrate enhanced reversible uptake of molecular oxygen. Coordination of the dioxygen molecule by the “active” complex occurs as exchange of the axial H_2_O or carboxyl oxygen to O_2_, occurring together with simultaneous formal intramolecular redox oxidation of Co(II) to Co(III) and the reduction of the charge of the dioxygen molecule to a bridging peroxide O_2_^2−^ ion.

The log  $K_{{{\text{O}}_{2} }}$ values are highest for the oxygenated forms of the heteroligand complexes with histidine, as their coordination sphere is formed by a chelating tridentate ligand (with imidazole, NH_2_, COO^−^ donors). The essential impact on the electron structure of the dioxygen bridge, and by that on reversibility of O_2_ uptake, is due to the first of the groups mentioned above. The two remaining amac ligands engaged in the mixed complexes (i.e. alanine and asparagine) were bidentate ligands. Even the potentially tridentate l-α-asparagine behaves as a bidentate ligand in the attainable pH range of around 9–10, illustrated in Table 1, which follows also from the previous reports concerning oxygen–free conditions. However, the reversibility of O_2_ uptake in the latter systems containing an axial imidazole, unequivocally indicates a much higher reversibility than that previously reported for Co(II)–amac systems in the absence of imidazole.

Chemically reasonable (positive) values of both [Co(II)], [amac], [Himid] equilibrium concentrations and hence, appropriate log $K_{{{\text{O}}_{2} }}$ values, could be attained only for limited Co(II):amac:Himid molar ratios, irrespective of the degree of equilibrium displacement towards oxygen adduct formation.

## Experimental

### Reagents

l-α-amino acids: asparagine, pure, Sigma Chemical Co., histidine, pure (≥99.0%), Fluka Chemie GmbH, alanine, pure, International Enzymes Limited; polymeric [Co(imid)_2_]_n_ complex prepared by A. Vogt, Faculty of Chemistry, University of Wrocław [27, 38, 45]; potassium nitrate (V), p.a., P.O.Ch. Gliwice; nitric (V) acid, p.a., P.O.Ch. Lublin; sodium hydroxide—0.5021 M solution determined by potassium hydrogen phthalate; acetone, p.a., P.O.Ch. Gliwice; oxygen pure medical (99.7–99.8%); argon, p.a. (99.999%) from Linde Gas (Poland).

### Apparatus

An isobaric laboratory set for volumetric and pH-metric measurements (see Additional file 3: Figure S3), composed of the following elements: a double-walled thermostated glass vessel of volume ca 80 mL, tightly closed with a silicon stopper and equipped with a burette nozzle supplying the 4 M HNO_3_; a combination pH glass electrode C2401, Radiometer (Copenhagen); a Radiometer Analytical 101 temperature sensor; a gas inlet tube (dioxygen) connected with the gas burette; outlet tube; a glass rode to hang a small glass vessel with the [Co(imid)_2_]_n_ polymer. A PHM 85 Precision pH Meter Radiometer (Copenhagen), a Fisherbrand FBC 620 cryostat, Fisher Scientific, an Electromagnetic Stirrer ES 21H (Piastów, Poland), an oxygen tank with reducing valve and a CO-501 Oxygen Meter, Elmetron (Zabrze, Poland) were also used. The following glass set was used to determine the imidazole released from the coordination sphere of the mixed complexes: suction flask, water suction pump, washer, Schott funnel POR 40 (see Additional file 4: Figure S4).

### Measurement procedures

#### Oxygenation reaction of the Co(II)–l-α-amino acid–imidazole systems

The thermostated vessel was filled with a solution containing an exactly weighted sample of chosen amino acid, so as to obtain a predicted Co(II)–amac–Himid ratio when adding the [Co(imid)_2_]_n_ polymer. Adjustment of the solution to constant ionic strength *I* = 0.5 M was achieved by means of potassium nitrate. The solution was topped up with water to 30 mL. A small glass vessel with 0.3 mmol of the [Co(imid)_2_]_n_ polymer (hence the same 0.6 mmol of imid) was hung from a glass rod over the solution surface. After the entire vessel reached a temperature close to 0 °C [decrease of temperature inhibits the irreversible oxidation of Co(II)], the initial pH and the initial volume level in the gas burette was read and the main experiment started by inserting the polymer into the sample. The current values of pH and dioxygen volume were noted in definite time intervals up to saturation. A rise in pH was observed along with a change in color from entirely colorless to brown or even dark-brown. At the end of oxygenation, which occurred when reaching pH ≈9 to 10, the solution was acidified to the initial pH with a small aliquot of 4 M nitric acid solution. This caused a partial discoloration of the solution and evolution of dioxygen. The volume of dioxygen evolved against the total volume of dioxygen bound served as a measure of reversibility of oxygenation.

#### Determination of reaction stoichiometry of dioxygen uptake in the Co(II)–l-α-amino acid–imidazole systems by the molar ratio method

For each system under study, a dependence plot of the number of bound O_2_ (mmol) against the *C*_L_/*C*_M_ ratio was prepared, where *C*_L_—total amac concentration, *C*_M_—total Co(II) concentration, which enabled the determination of stoichiometry of dioxygen uptake.

#### Confirmation of the coordination mode of the central ion by determination of the number of imidazole released from the coordination sphere of the Co(II)–l-α-amac–imidazole–O_2_ system

Exactly weighed samples of amino acid and the [Co(imid)_2_]_n_ polymer were placed into a washer so as to attain a molar ratio of Co(II):l-α-amac: imidazole = 0.3: 0.9: 0.3 (mmol). The washer immersed in ice was filled with 2 mL of argonated water and then, during 15 min, the forming “active” complex was argonated continuously. After 10 min, argonation was changed to oxygenation. The final content of the washer, the freshly formed dioxygen complex, was quantitatively added to a Schott funnel previously filled with oxygenated acetone. The oxygen complex, insoluble in water, precipitated as a dark brown solid. At the moment a water suction pump was connected to the Schott funnel. Acetone was filtered off together with the water containing the imidazole released along with oxygen complex formation. The filtrate obtained was titrated potentiometrically with nitric acid. All the steps of experiment were carried out at temperature close to 0 °C.

#### Calculations of equilibrium concentrations of Co(II), amac and Himid as well as evaluation of the equilibrium $K_{{{\text{O}}_{2} }}$ constants

The calculations were performed by means of a Mathcad 13 computer program [46]. The mass balance non–linear equation system was solved by the Levenberg–Marquardt method [47, 48], which enables a faster convergence of the solutions than the Gauss–Newton iteration. Such effect is due to the introduction of an additional *λ* parameter to the Gauss–Newton iteration formula, which corrects the appropriate direction of the procedure depending on whether the solutions go close to or far from the convergence series.

The procedure used the corresponding equilibrium concentrations [M], [L], [L′] (where: [M] = [Co(II)]), which were the searched unknown quantities *x*_1_, *x*_2_, *x*_3_ of the following system:

1 $$\begin{aligned} f_{ 1} (x_{ 1} ,x_{ 2} ,x_{ 3} ) = \, 0 \hfill \\ f_{ 2} (x_{ 1} ,x_{ 2} ,x_{ 3} ) = \, 0 \hfill \\ f_{ 3} (x_{ 1} ,x_{ 2} ,x_{ 3} ) = \, 0 \hfill \\ \end{aligned}$$

The solution vector of the system:

2 $$X = \left[ {\begin{array}{*{20}c} {x_{1} } \\ {x_{2} } \\ {x_{3} } \\ \end{array} } \right]$$

follows Newton’s formula:

3 $$X_{{{\text{i}} + 1}} = X_{\text{i}} - \, \left( {F^{\prime} \, \left( {X_{\text{i}} } \right)^{ - 1} \cdot F\left( {X_{\text{i}} } \right)} \right)$$

after an appropriate initial estimation of the *X*_0_ vector. The function vector is:

4 $$F (X) = \left[ {\begin{array}{*{20}c} {f_{1} (x_{1} ,x_{2} ,x_{3} )} \\ {f_{2} (x_{1} ,x_{2} ,x_{3} )} \\ {f_{3} (x_{1} ,x_{2} ,x_{3} )} \\ \end{array} } \right]$$

whereas the matrix of derivatives, i.e. Jacobi matrix, is:

5 $$F^{\prime}(X) = \left[ {\begin{array}{*{20}c} {\frac{{\partial f_{1} }}{{\partial x_{1} }}\quad \frac{{\partial f_{1} }}{{\partial x_{2} }}\quad \frac{{\partial f_{1} }}{{\partial x_{3} }}} \\ {\frac{{\partial f_{2} }}{{\partial x_{1} }}\quad \frac{{\partial f_{2} }}{{\partial x_{2} }}\quad \frac{{\partial f_{2} }}{{\partial x_{3} }}} \\ {\frac{{\partial f_{3} }}{{\partial x_{1} }}\quad \frac{{\partial f_{3} }}{{\partial x_{2} }}\quad \frac{{\partial f_{3} }}{{\partial x_{3} }}} \\ \end{array} } \right]$$

(*F*’(*X*))^−1^ in Eq. (3) denotes the inverted Jacobi matrix.

In the mass balance system all the ligand (both amac and Himid) protonation constants as well as the complex–formation constants with Co(II) were known from the previous reports [37, 45]. In cumulative form the formation constants may be written as:

6 $$\beta_{mll^\prime h} = {\raise0.7ex\hbox{${[{\text{M}}_{m} {\text{L}}_{l} L^\prime_{l\prime } H_{h} ]}$} \!\mathord{\left/ {\vphantom {{[{\text{M}}_{m} {\text{L}}_{l} L\prime_{l\prime } H_{h} ]} {[{\text{M}}]^{m} [{\text{L}}]^{l} [{\text{L}}^\prime ]^{l^\prime } [{\text{H}}]^{h} }}}\right.\kern-0pt} \!\lower0.7ex\hbox{${[{\text{M}}]^{m} [{\text{L}}]^{l} [{\text{L}}^\prime ]^{l^\prime } [{\text{H}}]^{h} }$}}$$

The functions used for the equation systems of l-α-alanine and l-α-asparagine were due to the fact that the mixed ML_2_L′ complex capable of dioxygen uptake (existing outside of the non-active mixed MLL′ complex) contains the sufficient three nitrogen donors in the coordination sphere, in accordance with Fallab’s “3 N” rule [49]:

7 $$f_{1} = C_{\text{M}} - Y[{\text{M]}} - \sum\limits_{l = 1}^{3} {\beta_{ml} [{\text{M]}}} \,[{\text{L}}]{\kern 1pt}^{l} - \sum\limits_{l' = 1}^{5} {\beta_{ml'} [{\text{M]}}} \,[{\text{L}}^{\prime}]{\kern 1pt}^{l'} - \sum\limits_{l = 1}^{2} {\beta_{mll'} [{\text{M]}}} \,[{\text{L}}]{\kern 1pt}^{l} [{\text{L}}^{\prime}] - 2C_{{{\text{O}}_{2} }}$$

8 $$f_{2} = C_{\text{L}} - Y_{1} [{\text{L]}} - l\sum\limits_{l = 1}^{3} {\beta_{ml} [{\text{M]}}} \,[{\text{L}}]{\kern 1pt}^{l} - l\sum\limits_{l = 1}^{2} {\beta_{mll'} [{\text{M]}}} \,[{\text{L]}}{\kern 1pt}^{l} [{\text{L}}^{\prime}] - 4C_{{{\text{O}}_{2} }}$$

9 $$f_{3} = C_{\text{L}^{\prime}} - Y_{2} [{\text{L}}^{\prime}] - l'\sum\limits_{l' = 1}^{5} {\beta_{ml'} [{\text{M]}}} \,[{\text{L}}^{\prime}]{\kern 1pt}^{l'} - \sum\limits_{l = 1}^{2} {\beta_{mll'} [{\text{M]}}} \,[{\text{L]}}{\kern 1pt}^{l} [{\text{L}}^{\prime}] - 2C_{{{\text{O}}_{2} }}$$

For l-α-histidine, the mixed not oxygen binding complex was a MLL′H species, in which the side group imidazole was protonated at the N3 nitrogen, thus the number of nitrogen atoms in the coordination sphere of the central ion was two, i.e. less than the minimum suggested by Fallab’s rule. However, as the number of nitrogen atoms was sufficient in the “active” complex ML_2_L′, capable of O_2_ was as follows:

10 $$f_{1} = C_{\text{M}} - Y[{\text{M]}} - \sum\limits_{l = 1}^{ 2} {\sum\limits_{h = 1}^{1} {\beta_{mlh} } } \,[{\text{M}}][{\text{L}}]{\kern 1pt}^{l} [{\text{H}}]{\kern 1pt}^{h} - \sum\limits_{l' = 1}^{5} {\beta_{ml'} [{\text{M]}}} \,[{\text{L}}^{\prime}]{\kern 1pt}^{l'} - \beta_{1210} [{\text{M}}][{\text{L}}]{\kern 1pt}^{2} [{\text{L}}^{\prime}] - \beta_{1111} [{\text{M}}][{\text{L}}]{\kern 1pt} [{\text{L}}^{\prime}][{\text{H}}]\, - 2C_{{{\text{O}}_{2} }}$$

11 $$f_{2} = C_{\text{L}} - Y_{1} [{\text{L]}} - l\sum\limits_{l = 1}^{ 2} {\sum\limits_{h = 0}^{1} {\beta_{mlh} } } \,[{\text{M}}][{\text{L}}]{\kern 1pt}^{l} [{\text{H}}]{\kern 1pt}^{h} - 2\beta_{1210} [{\text{M}}][{\text{L}}]{\kern 1pt}^{2} [{\text{L}}^{\prime}] - \beta_{1111} [{\text{M}}][{\text{L}}]{\kern 1pt} [{\text{L}}^{\prime}][{\text{H}}] - 4C_{{{\text{O}}_{2} }}$$

12 $$f_{3} = C_{\text{L}^{\prime}} - Y_{2} [{\text{L}^{\prime}]} - l'\sum\limits_{l' = 1}^{5} {\beta_{ml'} [{\text{M]}}} \,[{\text{L}^{\prime}]}{\kern 1pt}^{l'} - \beta_{1210} [{\text{M}}][{\text{L}}]{\kern 1pt}^{2} [{\text{L}^{\prime}]} - \beta_{1111} [{\text{M}}][{\text{L}}]{\kern 1pt} [{\text{L}^{\prime}][H]} - 2C_{{{\text{O}}_{2} }}$$

where: *C*_M_—total concentration of the metal: Co(II), *C*_L_—total concentration of the l-α-amino acid, $C_{{L^{\prime}}}$—total concentration of imidazole, $C_{{{\text{O}}_{2} }}$—concentration of the oxygen adduct, *β*_*ml*_—summary stability constants of the Co(II)–l-α-amino acid complexes, *β*_*ml’*_—summary stability constants of the Co(II)–imidazole complexes, *β*_*mll’*_—summary stability constants of the mixed Co(II)– l-α-alanine/asparagine–imidazole complexes, *β*_1210_, *β*_1111_—summary stability constants of the mixed Co(II)–l-α-histidine–imidazole complexes.

The hydrolyzed Co(II) aqua-ion and the protonated (not complexed) ligand forms were considered in expressions:

$$\begin{aligned} Y &= 1 + \left( {{\text{1}}/K_{{{\text{OH}}}} [{\text{H}}]} \right) \\Y_{1} &= 1 + \beta _{{{\text{LH}}}} [{\text{H}}] + \beta _{{{\text{LH2}}}} [{\text{H}}]^{{\text{2}}} \,&{\text{for}} \,{\textsc{{l}}}\text{-}\alpha{\text{-alanine and}}\,{\textsc{{l}}}\text{-}\alpha {\text{-asparagine}};\hfill \\ {\text{Y}}_{{\text{1}}}&= {\text{ 1 }} + \beta _{{{\text{LH}}}} [{\text{H}}] + \beta _{{{\text{LH2}}}} [{\text{H}}]^{{\text{2}}} + \beta _{{{\text{LH3}}}} [{\text{H}}]^{{\text{3}}}\;&{\text{for}}\, {\textsc{{l}}}\text{-}\alpha\text{-histidine} \hfill\\ Y_{2} &= 1 + \beta _{{{\text{L}}^{\prime } {\text{H}}}} \left[ {\text{H}} \right] \hfill\end{aligned}$$

where: *K*_OH_—hydrolysis constant of tshe Co(II) aqua-ion = 10^−9.8^ [50], *β*_LH_, *β*_LH2_, *β*_LH3—_summary (overall) protonation constants of the l-α-amino acid, *β*_L’H_—protonation constant of imidazole.

It is noteworthy that solving the nonlinear equation system at very erroneous initial estimations may lead to quite different results or lack of convergence. However, in the case of the systems under study, the solutions [M], [L] and [L′] are not allowed to be negative numbers and they should be found within the limits of zero and the total concentrations *C*_M_, *C*_L_, *C*_L′_. This makes it possible to reject the solutions without a chemical meaning.

The used summary protonation constants of l-α-amino acids and imidazole, the stability constants of the primary Co(II)–amac, Co(II)–Himid complexes, as well as the stability constants of the heteroligand Co(II)–l-α-amino acid–imidazole complexes have been determined previously in the same medium and the same ionic strength as in the present work (KNO_3_, *I* = 0.5) [37, 45]. The only different parameter was the temperature: 25.0 °C, instead of 0–1 °C. The lack of data due to the lower temperature is usually caused by lowered sensibility of the glass electrodes. Nevertheless, the systematic error of the stability constants recently used could be estimated on the basis of corresponding literature data as 0.1–0.2 in logarithm [51].

The obtained equilibrium concentrations [M], [L], [L′] were needed to calculate the $K_{{{\text{O}}_{2} }}$ constant. In the present reaction scheme, the first step corresponded to formation of the “active” complexes:

13 $${\text{Co}}\left( {\text{imid}} \right)_{ 2} + {\text{ 2 Hamac }} + {\text{ H}}_{ 2} {\text{O}} \to {\text{Co}}\left( {\text{amac}} \right)_{ 2} \left( {\text{Himid}} \right)\left( {{\text{H}}_{ 2} {\text{O}}} \right) \, + {\text{ Himid}}$$

Consecutively the “active” complex takes up dioxygen by forming the dimeric oxygen adduct:

14 $$2 {\text{ Co}}\left( {\text{amac}} \right)_{ 2} \left( {\text{Himid}} \right)\left( {{\text{H}}_{ 2} {\text{O}}} \right) \, + {\text{ O}}_{ 2} \to \, \left[ {{\text{Co}}\left( {\text{amac}} \right)_{ 2} \left( {\text{Himid}} \right)} \right]_{ 2} {\text{O}}_{ 2}^{ 2- } + {\text{ 2 H}}_{ 2} {\text{O}}$$

By treating the O_2_ uptake as a reversible reaction:15the equilibrium constant may be calculated from the formula:

16 $$K_{{{\text{O}}_{2} }} = \frac{{[{\text{O}}_{2} \,{\text{adduct}}]}}{{[ \, {\text{"active"}}{\text{ complex}}]^{2} [{\text{O}}_{2} ]}}$$

where [O_2_ adduct]—equilibrium concentration of this part of the oxygen adduct, in which dioxygen was bound reversibly. The value was found by using the percentage of reversibility of O_2_ uptake, that is to say by rejecting the part of O_2_ adduct, in which the metal undergoes irreversible oxidation to Co(III) during the experiment. The equilibrium [O_2_] concentration was calculated on the basis of table data of dioxygen solubility in water [52].

According to Henry’s law, if the experiment proceeds at the same temperature but at decreased pressure, the volume of gas dissolved in water (or in a diluted solution) is proportionally lower. Under the experimental conditions we have:

$$V_{{{\text{O}}_{ 2} }} = V_{\text{g}} \cdot f = V_{\text{g}} \cdot p_{{{\text{O}}_{ 2} }} / 7 60$$

where: *V*_g_ = 0,04758 mL—table value of dioxygen solubility in 1 L of water, at temperature 1 °C under normal pressure 1013 × 10^5^ Pa. $p_{{{\text{O}}_{ 2} }}$—partial pressure of dioxygen in the gas burette.

The $V_{{{\text{O}}_{ 2} }}$ value gives the [O_2_] concentration after adjustment to the number of mmoles of O_2_ dissolved in 1 L of the solution.

## Additional files

**Additional file 1: Figure S1.** Determination of stoichiometry of the O_2_ uptake by the molar ratio method in the Co(II) – amac – Himid – O_2_ systems. L-α-amino acid (amac) = (a) alanine, (b) asparagine, (c) histidine. *C*_L_ – total concentration of amac, *C*_Co_ – total concentration of Co(II). Mmole O_2_ – number of mmol of dioxygen taken up. All the samples contained 0.3 mmol of Co(imid)_2_ in 30 mL of solution.

**Additional file 2: Figure S2.** Titration curve of the water-acetone filtrate obtained when the dioxygen adduct formed in aqueous solution precipitated in acetone. Co(II) : amac : Himid at a molar ratio of 0.3 : 0.9 : 0.3 (mmol); L-α-amino acid (amac) = (a) alanine, (b) asparagine, (c) histidine.

**Additional file 3: Figure S3.** Laboratory set for pehametric – volumetric measurements.

**Additional file 4: Figure S4.** Laboratory set for determination of imidazole released from the coordination sphereof Co(II): (a) initial preparation, (b) collection of the filtrate.

## Abbreviations

imid
Himid
amac: amino acid (α-amine group non-protonated, carboxyl group deprotonated)
Hamac: amino acid (α-amine group protonated, carboxyl group deprotonated)
